# Hospital Residents and Economy

**Published:** 1907-04-20

**Authors:** 


### Resident Medical Officers' Department.

### HOSPITAL RESIDENTS AND ECONOMY.

It is doubtless true that house physicians, and resident medical officers generally, often prescribe expensive drugs without considering whether cheaper medicines might not prove equally effective. This is especially the case in the out-patient and casualty departments of large hospitals. In the wards, the treatment is largely under the control of the visiting staff, and we do not propose to criticise from an economical point of view the prescriptions of the leading members of the profession. What we are here concerned with, is the indiscriminate ordering of expensive drugs by junior staff officers, often in large doses, and often for long periods of time. This common form of extravagance is not so much due to carelessness, as to ignorance of the relative cost of medicines. Newly-qualified practitioners, with no experience of general practice and of drug bills, have usually the vaguest ideas of what medicines cost. As students they may have been taught to avoid unnecessary extravagance with dressings and bandages, but we do not know of a single teacher of materia medica or therapeutics who, in his teaching of these subjects, makes any point of economy in the use of medicines, or even indicates to his pupils the fact that some drugs are more costly than others, and that some are very costly indeed. These may be sordid details, unworthy of the attention of a consulting physician, but to the general practitioner they are distinctly important, and the sooner he grasps them the better.

We do not wish for a moment to suggest that economy in drugs should be pushed to absurd limits. There are certain absolutely indispensable remedies which, however costly, must be prescribed in full doses, if the patient is to have the best, or, indeed, the only treatment for his disease. Potassium iodide, for example, and antidiphtheritic serum must often be given in large quantities, without regard to the expense incurred. Economy in such cases is impossible.

But, on the other hand, there are many chronic diseases, frequently met with in out-patient practice, which need not be treated, as they often are, and for many months, with large doses of expensive drugs. If it were certain that proportionate benefit was derived, the case would be quite different. It would be quite justifiable, for instance, for a house physician to give pounds' worth of iodides and guaiacol to every patient with osteo-arthritis, if he were convinced that by these means only the patient would be really relieved. But iodides are not a specific for chronic rheumatic affections, as they are for tertiary syphilis and for many cases of actinomycosis. They occasionally seem to do some little good, it is true; and they are recommended, among other measures, by the best authorities; but the disease is practically incurable, and general practitioners, who cannot afford to supply such costly medicines, are often successful in relieving their patients' sufferings with cheaper remedies.

These are only cited as examples to illustrate our point. We cannot here mention all the expensive drugs which are constantly ordered in routine fashion for casualty patients, nor can we contrast the prices of the various alternative remedies which may be used for the same diseases. Details of this sort can be obtained from the hospital dispenser, or may be compiled from the text-books with the aid of a druggist's price-list. What we wish to convey is, that resident medical officers can often save their hospitals large sums of money by remembering that drugs vary greatly in price, and that from the cheaper ones they may still obtain good results in treatment. Hospital pharmacopœias are in the main compiled with a view to economy, but they do not meet all cases, and it is quite possible to depart from them and yet avoid extravagance.

With regard to diet and extras for in-patients, the resident staff are usually allowed the same latitude as is given to them in the case of the treatment of out-patients. Here also they can exercise economy, and, at the same time, do the best for their patients.

It is, of course, very tempting for a humane house surgeon to order delicacies for poor patients who are accustomed to semi-starvation and the coarsest of food. He feels that their stay in hospital should be made as pleasant as possible. But while taking care to provide everything that is essential for their comfort and their proper treatment, he should remember that hospital incomes are limited, and that every luxury ordered for one case may mean so much the less money for the bare necessities of another case. The ordering of diets is nearly everywhere left largely to the discretion of the resident staff, who should be careful to remember their responsibilities in this matter. Extras, including alcohol, soon mount up, and very slight extravagance in this direction greatly increases the cost per bed of a hospital. In some hospitals where funds are low and the pinch of competition is felt, alcohol has to be signed for by the visiting staff. This rule is naturally often evaded, and we believe that it is a poor way out of the difficulty. It would be better to put the matter plainly before each house officer on his appointment, and to leave the honourable fulfilment of the governors' wishes in his hands. Any want of confidence in their loyalty and good sense is keenly resented by resident medical officers, and there is little doubt that the work of the hospital is carried out most efficiently and most economically in those institutions where pleasant relations exist between governors and staff, and where the resident officers enjoy a reasonable authority and are not subjected to unnecessary interference and dictation.

